# The Magnitude and Determinants of Suboptimal Child Spacing Practices Among Women of Childbearing Age in Ethiopia: A Systematic Review and Meta-Analysis

**DOI:** 10.1089/whr.2024.0179

**Published:** 2025-03-25

**Authors:** Abebaw Alamrew, Mulat Ayele, Eyob Shitie Lake, Getinet Kumie, Haimanot Hailu, Aynalem Yetwale, Tadele Emagneneh, Chalie Mulugeta

**Affiliations:** ^1^ Department of Midwifery, College of Health Sciences, Woldia University, Woldia, Ethiopia.; ^2^ Department of Medical Laboratory Science, College of Health Sciences, Woldia University, Woldia, Ethiopia.; ^3^ Department of Information Technology, School of Computing, Institute of Technology, Woldia University, Woldia, Ethiopia.

**Keywords:** interpregnancy interval, short birth interval, systematic review, meta-analysis, women, Ethiopia

## Abstract

**Background:**

Short birth intervals (SBI), also known as suboptimal childbirth intervals, are frequent and have detrimental effects on both mother and child health. There is limited national data except for small-scale studies on the prevalence and contributing factors of SBI practices in Ethiopia. We did this review to find the pooled prevalence of suboptimal birth spacing and its contributing factors among Ethiopian women of reproductive age.

**Methodology:**

This study followed the PRISMA guideline. Articles were found using MEDLINE/PubMed, Scopus, Hinari, Google Scholar, and Web of Science. Subgroup analysis was used to look for heterogeneity evidence. *I*^2^ statistics and funnel plots with the Egger test were used to assess the studies’ heterogeneity and publication bias.

**Results:**

In total, 19 studies were included in this meta-analysis with a sample size of 11,674. The pooled prevalence of SBI was 50.29% (95% confidence interval [CI], 43.18, 57.40). Rural residency (adjusted odds ratio [AOR] = 2.13; 95% CI: 1.19, 3.07), age at first marriage less than 18 (AOR = 1.94; 95% CI: 1.34, 2.54), women with no formal educational status (AOR = 3.39; 95% CI: 2.59, 4.19), no contraceptive use (AOR = 4.20; 95% CI: 2.84, 5.56), duration of breastfeeding less than 24 months (AOR = 3.44; 95% CI: 1.64, 5.25), female sex of the index child and survival (death) of the index child (AOR = 2.34; 95% CI: 1.53, 3.15), and (AOR = 2.17; 95% CI: 1.02, 3.31), respectively, were the main determinants of suboptimal child spacing.

**Conclusion:**

The pooled prevalence of suboptimal child spacing practices in Ethiopia was found to be high almost half of the births were suboptimal.

## Background

Birth interval is the time between two consecutive deliveries that is expressed in months.^1^ The World Health Organization suggests a minimum of 24 months between becoming pregnant or 33 months between two consecutive births to lower the risk of poor outcomes for the health of mothers, perinatal, and infants.^2^ Suboptimal childbirth interval or short birth interval (SBI), defined as an interval of less than 33 months between two subsequent births, between 33 and 59 months is considered the optimum birth interval and greater than 59 months is the long birth interval.^3–5^

Countries with low- and lower-middle incomes frequently have high rates of fertility.^6^ SBIs are brought on due to this high fertility rate and are an important health issue, particularly in low- and middle-income nations. Studies have shown that the prevalence of SBIs in sub-Saharan Africa is still quite high, ranging from 23% to 56%.^4^ In Ethiopia, the median birth interval is 38 months, as reported in national surveys such as the Ethiopian Demographic and Health Survey 2016 (EDHS 2016). Although this study does not specifically assess the prevalence of SBIs across the country, it is important to recognize that the national median suggests many women achieve adequate spacing between births. However, the occurrence of shorter-than-recommended intervals remains an important issue that requires attention.^7^

Studies showed that suboptimal birth intervals are associated with an increased risk of certain adverse perinatal outcomes, stillbirth,^6,8^ low birth weight,^1,6,9–16^ early neonatal mortality,^3,6,8,14,17^ perinatal mortality,^18,19^ preterm delivery,^6,9–12,20–22^ small for gestational age,^9,12,14,21^ low Apgar score,^9,11,14^ postneonatal mortality,^18^ infant mortality,^3,17,18,21,23,24^ child mortality^18,23,25^ under-five mortality (U5M),^3,17,18^ and also increased under nutritional outcomes (stunting and under weight).^1^

Studies showed that suboptimal birth intervals are associated with an increased risk of certain adverse maternal outcomes maternal death,^26^ obstructed labor,^6^ maternal hemorrhage,^6,9,26–28^ fetal malposition,^6,14^ maternal infection^6,26^ and maternal hospitalization,^6^ premature rupture of membranes,^6,9,14,29^ preeclampsia,^6,14^ anemia,^9,26,27^ higher risk for failure of trial of vaginal birth after cesarean section,^9^ scar dehiscence in postcesarean pregnancies,^27^ placenta previa,^30^ and abruption placenta.^30^

The UN’s sustainable development goal (SDG) goal target was to achieve a maternal mortality ratio of less than 70 per 1,00,000 live births, neonatal mortality of at least as low as 12 per 1,000 live births, and under-5 mortality of at least as low as 25 per 1,000 live births.^31–32^ Ethiopia, as a member country, has adopted the goals and has been giving great attention and efforts to achieve the target by 2030. Despite the decline in maternal, neonatal, and under-5 mortality in Ethiopia, the mortality is still high and not close to the SDG target.^31^ Yet, 1 in 267 women dies of pregnancy-related causes, 1 in 20 children dies before reaching the age of 5, 1 in 28 infants dies before reaching 1 year of age, and 1 in 38 newborns dies within their first 4 weeks of life, which are still too high.^33,34^ The objective of this systematic review and meta-analysis was to figure out the pooled prevalence of suboptimal birth spacing and its contributing factors among Ethiopian women who are of reproductive age. Policymakers and program designers may use the findings of this study as a starting point to develop evidence-based child spacing interventions. It will also be extremely important for researchers in the future who are interested in related fields.

## Material and Methods

### Review question

The review questions of this systematic review and meta-analysis were “What are the pooled magnitude and factors associated with suboptimal birth spacing (short birth interval) in Ethiopia?”

### Outcomes of the review

The primary outcome was pooled magnitude of suboptimal birth spacing (SBI) in Ethiopia and the secondary outcome of the review was factors associated with suboptimal birth spacing (SBI) in Ethiopia.

#### Birth interval

It is the interval between two successive deliveries and it is measured in months. According to the World Health Organization (WHO) recommendation, there should be at least 33 months between two consecutive live births. The inter-birth interval of *<*33 months is considered an SBI, between 33 and 59 months is considered the optimum birth interval, and greater than 59 months is the long birth interval.^35^

### Development of the review method

**FIG. 1.**
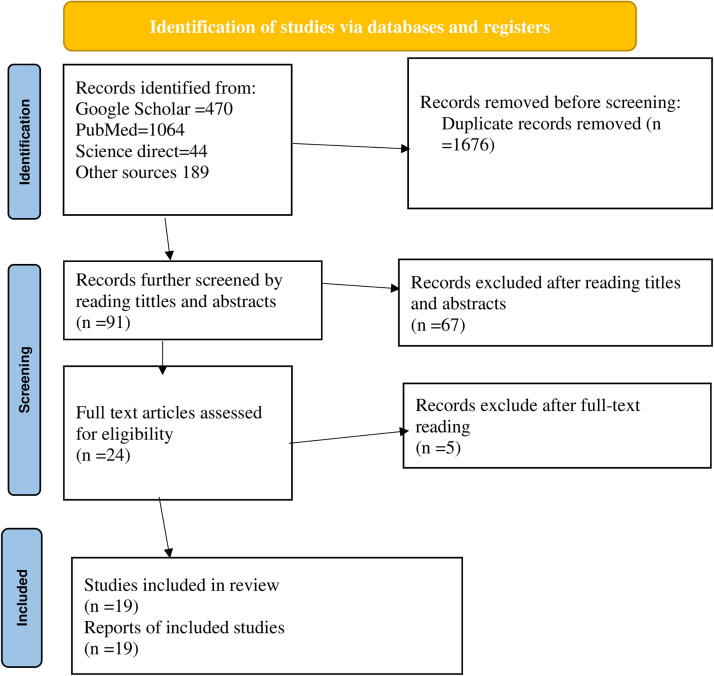
PRISMA flowchart showing the included searches for systematic review and meta-analysis entitled magnitude of suboptimal child spacing practices and its determinants among women of childbearing age in Ethiopia.

The Preferred Reporting Elements for Systematic Reviews and Meta-Analyses Protocols (PRISMA-P) 2020 statement served as the base for this systematic review’s methodology (Supplementary Data S1), and all of the PRISMA-P checklist’s items were covered.^36^ The PRISMA flowcharts, which illustrate the study selection procedure from the first identified records to the included studies, were recorded in the results (Fig. 1). The International Prospective Register of Systematic Reviews (PROSPERO) issued a registration number for this systematic review and meta-analysis CRD42023491553.

### Searches

EMBASE, PubMed, CINAHL, Google Scholar, MEDLINE, and African Journals Online were used by using search terms (Supplementary Data S2). Articles published in English and accessible for title, abstract, and full-text screening were included. Additionally, the reference lists of all included full-text articles were reviewed to identify further relevant studies for consideration. In addition, the references list of previous review articles were also scoped to identify any missing articles for consideration.

### Search date

This systematic review initial search was started on November 20, 2023, and the protocol was registered on December 17, 2023. This systematic review and meta-analysis includes all studies published in English and published in Ethiopia until December 15, 2023.

### Study selection and eligibility criteria

#### Inclusion criteria

**Participants**: This review included studies conducted on prevalence and factors associated with short birth intervals in Ethiopia

**Setting**: Studies conducted both at the community or institutional level.

**Study design:** Both cross-sectional and case-control studies were included in this review. Although cross-sectional studies provided magnitude estimates for suboptimal birth spacing, case-control studies focused on identifying the determinants of suboptimal birth spacing.

**Article**: Articles that include short birth intervals as the outcome variable.

**Time frame**: All studies irrespective of time of data collection period and publication year.

**Publication types**: Journal articles and studies published only in the English language were included in the review.

#### Exclusion criteria

Studies that did not report the outcome variable of this review, repetitive publications, and studies that did not specify the study population, conference papers, reviews, abstracts, and study protocols were excluded from this systematic review and meta-analysis.

### Data extraction

Five authors (A.A., M.A., E.S., G.K., and A.Y.) independently reviewed the database search results and excluded articles based on title and abstract screening using the inclusion and exclusion criteria. Articles with unclear topic details were sent for full-text review. The full text of the remaining papers was sourced and independently evaluated to determine if all inclusion and exclusion criteria were met. Following this process, the four researchers met and discussed each article to determine if it should be included in the systematic review. Any disagreements were sent to a sixth and seventh author to independently assess the article/s for inclusion and to make the final decision. The data abstraction form contains the first author’s name, publication year, study setting/region, study period, study design, sample size, and adjusted odds ratio (AOR) with a 95% confidence interval (CI) for significant risk factors of sunlight exposure practice.

### Risk of bias (quality) assessment

Four review authors (A.A., C.M., M.A., and E.S.) independently appraised the papers selected for inclusion for quality. The authors resolved disagreements by discussion or with the consultation of the fifth and seventh review authors (G.K. and A.Y.) in case of persisting disagreement. The authors involved content experts to judge any other flaws that could be overlooked by nonexperts and the included studies were assessed using the Newcastle–Ottawa scale checklist^37^ and all the included studies were high quality (Supplementary Data S3).

### Data synthesis

The data from the results of each included study were extracted into defined data extraction spreadsheets. Quantitative articles were pooled in a statistical meta-analysis using the Meta-XL. The effect sizes and their 95% CIs were calculated for analysis. Heterogeneity was assessed using the *I*^2^ statistic, and a random-effects model was applied if significant heterogeneity (*I*^2^ > 50%) was present.

### Sensitivity and subgroup analyses

Sensitivity analyses were conducted to assess the robustness of the findings by excluding studies. Subgroup analyses were performed based on factors of the study region.

### Data processing and analysis

The meta-analysis was conducted using STATA 11 software. A flowchart of the whole process, protocol of data extraction, and forest plots were used to present the findings of this meta-analysis. The *I^2^* test statistics and Egger’s test were used to test heterogeneity and publication bias, respectively.

## Results

### Description of the studies

This systematic review and meta-analysis included 19 studies published in Ethiopia with the outcomes of magnitude and its associated factors of suboptimal child spacing practices. The total number of study participants included in this review was 11,676 which varied among studies, including 150^38^ and 1,262^35^ we included five studies from the Amhara region,^35,38–41^ five from the SSNP,^42–46^ six studies from the Oromia region,^47–52^ one from Harer,^33^ one from the Tigray region^53^ and one from the Somalia region^54^ (Table 1).

**Table 1.** Description of the Included Studies on the Magnitude of Suboptimal Child Spacing Practices and Its Associated Factors Among Women of Childbearing Age in Ethiopia

Author name, year	Study region	Study area	Study design	Sample size	Study participants	Prevalence of suboptimal child spacing practices (%)	Factors associated with sub-optimal child spacing practices
Gedefaye Nibret, et al., 2021	Amhara	Rural Farta woreda, South Gondar zone	Case-control	150	150		Nonuse of contraceptives, unintended pregnancy, duration of breastfeeding,
Amare Genetu, et al., 2019	Amhara	Debremarkos East Gojjam zone	Institution-based cross-sectional	418	411	40.9	Nonuse of contraceptives, duration of breastfeeding, Survival status of index child, sex of indexed child,
Mastewal Belayneh, 2020	Amhara	Dembecha district West Gojjam zone	Community-based cross-sectional	895	880	43.4	Nonuse of contraceptives, rural residency, age at first marriage
Abebaw Addis, et al., 2023^35^	Amhara	Dabat district south Gondar zone	Community-based cross-sectional	1,393	1,262	30.59	Rural residency
Habtamu Shimels et al., 2020^39^	Amhara	Dessie city administration south Wollo	Case-control	678	654		Sex of indexed child, duration of breastfeeding, unintended pregnancy,
Musa Mohammed, 2022	Oromia	Mieso agro-pastoralist district,	Community-based cross-sectional	490	484	56	Nonuse of contraceptives, age at first marriage, duration of breastfeeding, educational status of the mother, status of index child, sex of indexed child
Seifadin Ahmed and Tesfaye Gobena, 2019	Oromia	Dodota Woreda, Arsi Zone	Community-based cross-sectional	660	647	51	Nonuse of contraceptives, rural residency, duration of breastfeeding, educational status of the mother, and history of short birth interval
Tewodros Yosef, et al., 2023^50^	Oromia	Gedeb Hasasa district, West Arsi Zone	Community-based cross-sectional	726	714	50.4	Rural residency, survival status of index child, sex of indexed child, duration of breastfeeding
Zenebu Begna, et al., 2013^49^	Oromia	Yaballo Woreda, Borena zone	Case-control	652	636		Educational status of the mother, nonuse of contraceptive, sex of indexed child
Dereje Tsegaye, et al., 2017^52^	Oromia	Illubabor zone	Community-based cross-sectional	826	811	51.2	Rural residency, survival status of index child, nonuse of contraceptive
Girma Bacha Ayane, et al., 2019	Oromia	Serbo Town Jimma Zone	Community-based cross-sectional	314	314	59.9	Educational status of the mother, nonuse of contraceptive, duration of breastfeeding, age at first marriage, sex of indexed child
Solomon Weldemariam, et al 2019	Tigray	Tselemti district	Community-based cross-sectional	803	806	23.3	Nonuse of contraceptives, unintended pregnancy, duration of breastfeeding
Samuel Yohannes, et al., 2011^42^	SNNP	Lemo Woreda	Community-based cross-sectional	811	552	57.6	Rural residency, nonuse of contraceptives, duration of breastfeeding
Desta Hailu and Teklemariam, 2016	SNNP	Arba Minch Zuria District	Case-control	636	636		Educational status of the mother, nonuse of contraceptive, sex of indexed child
Alemu Workineh et al., 2020	SNNP	West Badwacho district, Hadyia Zone	Community-based cross-sectional	626	626	60.4	Educational status of the mother, nonuse of contraceptive, sex of indexed child, age at first marriage
Yakob Lencha and Fentaw Wassie, 2022	SNNP	Mareka District, South Ethiopia	Community-based cross-sectional	768	703	58.5	Educational status of the mother, rural residency, nonuse of contraceptives,
Biruk Meskele, et al., 2023^44^	SNNP	Sodo Zuria District, Wolaita zone	Community-based cross-sectional	609	601	61.7	Educational status of the mother
Sultan Feyiso, 2021	Harer	OdaBultum Woreda West Hararghe Zone	Institution-based cross-sectional	404	404	59.9	Educational status of the mother, age at first marriage, duration of breastfeeding
Abdurahman Kedir, et al., 2021	Somalia	Jigjiga city administration	Case-control	388	388		Educational status of the mother, nonuse of contraceptive, sex of the indexed child, survival status of the index child

SNNP, Southern Nations, Nationalities, and Peoples' Region.

### Prevalence of suboptimal birth spacing (SBI) among reproductive age group women in Ethiopia

**FIG. 2.**
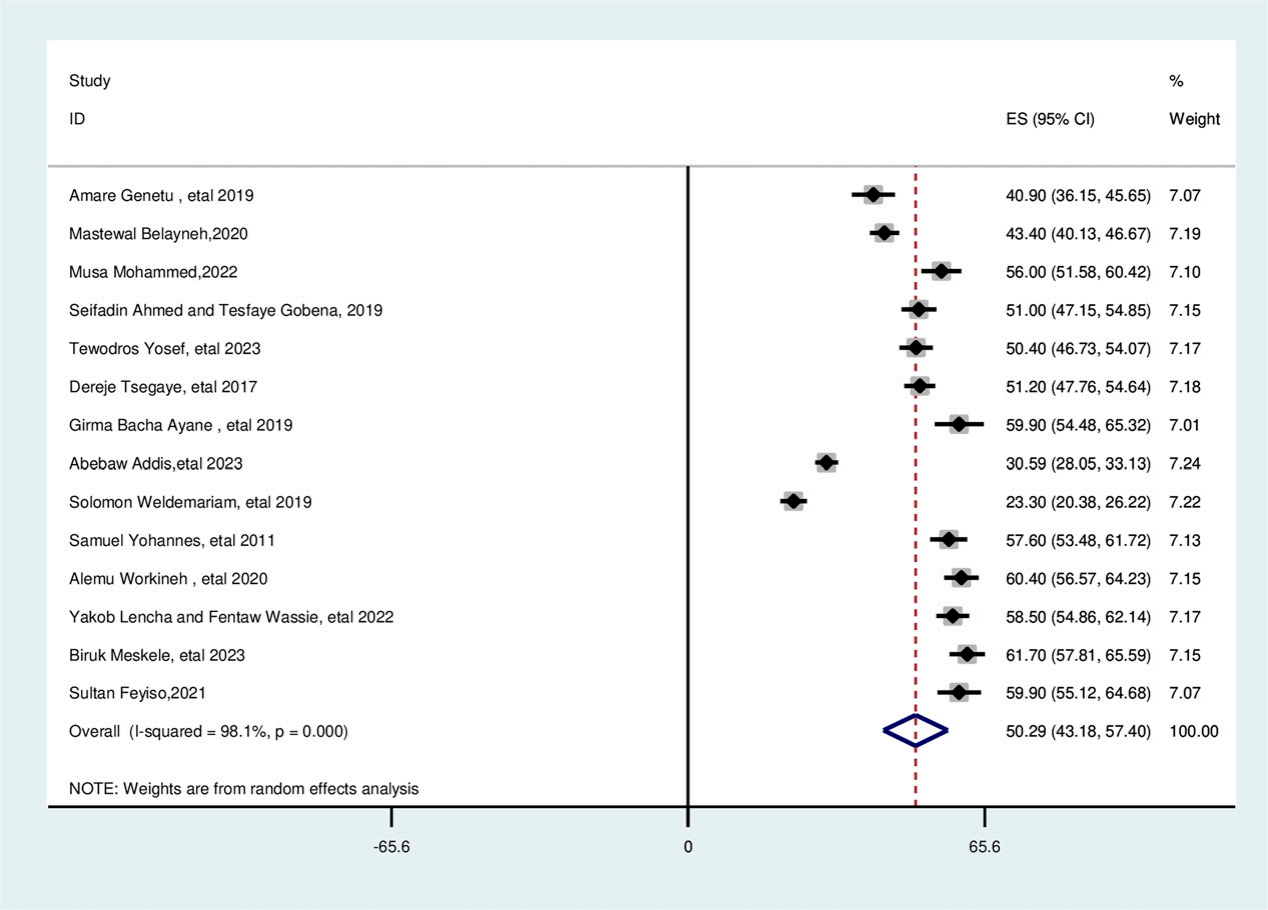
Pooled prevalence of suboptimal birth spacing among reproductive age women in Ethiopia.

In this systematic review and meta-analysis, the pooled prevalence of suboptimal birth spacing (SBI) among reproductive age group women was 50.29% (95% CI, 43.18–57.40). There was high heterogeneity (*I*^2^ = 98.1%, *p* = 0.000) (Fig. 2). Subgroup analysis showed this high heterogeneity was observed in studies conducted in the Amhara region and southern region, which showed the prevalence of suboptimal birth spacing of 38.21% (95% CI, 29.31, 47.11 (*I*^2^ = of 95.1%) and 59.57 (95% CI, 57.64, 47, 61.50), respectively. In this analysis of the Amhara and Southern regions, we observed notable differences in the factors associated with SBIs. For Amhara, key factors include the lack of contraceptive use (AOR = 7.76) and breastfeeding duration of less than 24 months (AOR = 2.35), which significantly contribute to higher odds of SBIs. Rural residence (AOR = 0.57) appears to reduce the likelihood of SBIs, and the age of marriage less than 18 (AOR = 1.65) also remains a risk factor. In the Southern Region, contraceptive use (AOR = 3.73) and breastfeeding duration of less than 24 months (AOR = 4.56) are strongly associated with SBIs. Unlike Amhara, rural residence (AOR = 2.38) and age of marriage less than 18 (AOR = 2.18) increase the odds of SBIs. Lack of formal education (AOR = 3.55) also plays a significant role. These findings suggest that regional differences, such as contraceptive use, education, and rural-urban residence, influence birth spacing patterns and should be considered in public health interventions (Supplementary Table S1).

### Factors associated with suboptimal birth spacing practice

This systematic review and meta-analysis revealed that rural residency, age of the mother at first marriage, educational status of the woman, not using contraceptives, duration of breastfeeding, sex of the indexed child, and survival status of the indexed child were the main determinants of suboptimal birth spacing practice in Ethiopia.

### Place of residence

**FIG. 3.**
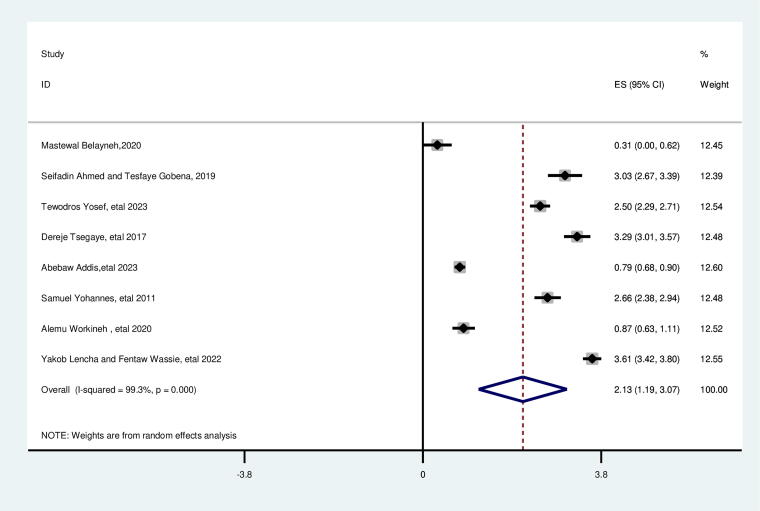
Pooled association between rural residency and suboptimal child spacing among reproductive age group women in Ethiopia.

The random effect analysis of the included studies revealed a significant association between place of residency and suboptimal birth spacing. The likelihood of SBI was 2.13 times higher for women living in rural regions (AOR = 2.13; 95% CI: 1.19, 3.07). This included study’s heterogeneity was found (*I*^2^ = 99.3%, *p* = 0.000), and the Egger test revealed no publication bias with a *p* value of 0.789 (Fig. 3).

### Age of mother at first marriage

**FIG. 4.**
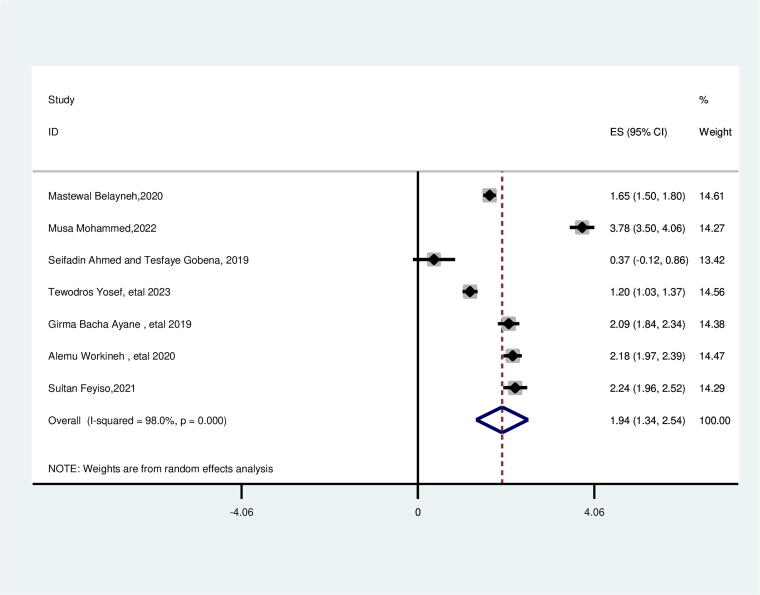
Pooled association between age at first marriage less than 18 and suboptimal child spacing among reproductive age group women in Ethiopia.

This review showed a significant association between the age of first marriage and suboptimal birth spacing. The likelihood of suboptimal child spacing was 1.94 times higher for women who were married before the age of 18 (AOR = 1.94; 95% CI: 1.34, 2.54). This included study’s heterogeneity was found to be higher (*I*^2^ = 98.0%, *p* = 0.000), and the Egger test identified no publication bias with a *p* value of 0.241 (Fig. 4).

### Educational status of the woman

**FIG. 5.**
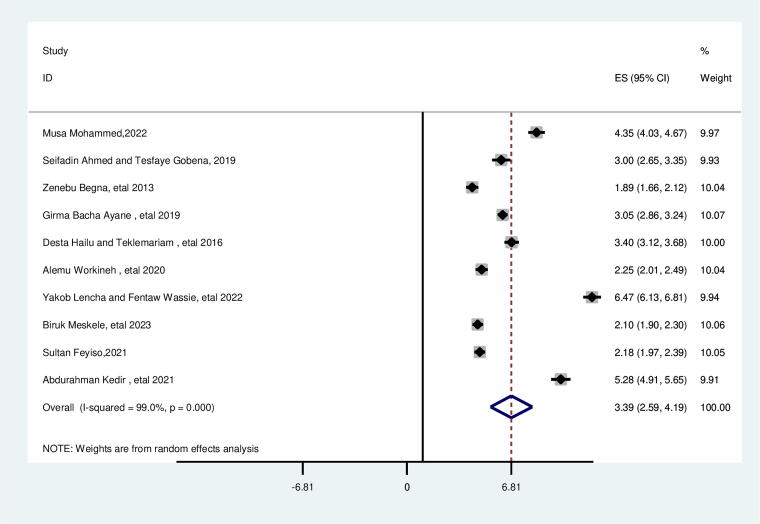
Pooled association between women with no formal educational status and suboptimal child spacing among reproductive age group women in Ethiopia.

In this review, the educational status of the woman had a significant association with suboptimal birth spacing. The likelihood of suboptimal child spacing was 3.39 times higher for women who were unable to read and write than their counterparts (AOR = 3.39; 95% CI: 2.59, 4.19). This included study’s heterogeneity was found to be high (*I*^2^ = 99.0%, *p* = 0.000), and subgroup analysis revealed that high heterogeneity occurred in a study conducted in Oromia and Southern regions and the Egger test identified no publication bias with a *p* value of 0.524 (Fig. 5).

### Contraceptive

**FIG. 6.**
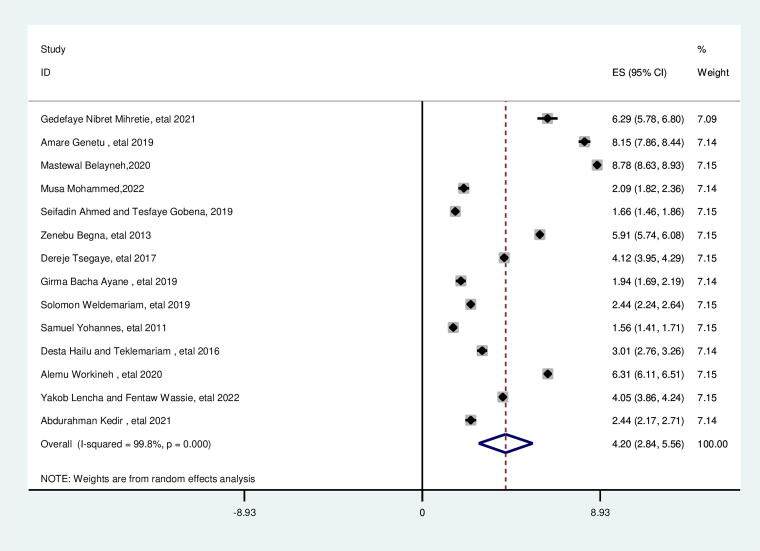
Pooled association between no contraceptive use and suboptimal child spacing among reproductive age group women in Ethiopia.

This review showed that suboptimal birth spacing is higher among women who are not using contraceptives than their counterparts. women who are not using contraceptives were nearly four times more likely to give birth less than 33 months than their counterparts (AOR = 4.20; 95% CI: 2.84, 5.56). This included study’s heterogeneity was found to be high (*I*^2^ = 99.8%, *p* = 0.000), and the Egger test identified no publication bias with a *p* value of 0.684 (Fig. 6).

### Duration of breastfeeding

**FIG. 7.**
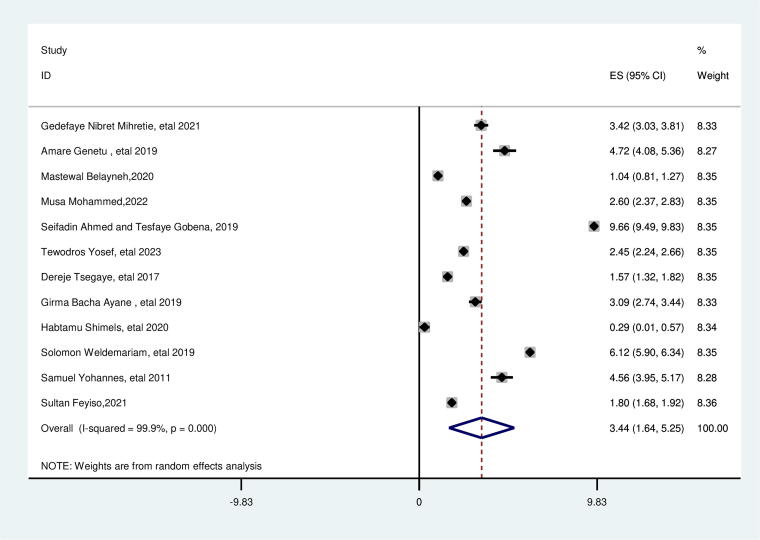
Pooled association between duration of breastfeeding less than 24 months and suboptimal child spacing among reproductive age group women in Ethiopia.

This review showed a significant association between the duration of breastfeeding and suboptimal birth spacing. In this study, women who breastfed their index child for less than 24 months had 3.44 times higher odds of experiencing suboptimal birth spacing compared to those who breastfed for 24 months or longer (AOR = 3.44; 95% CI: 1.64, 5.25). This included study’s heterogeneity was found high (*I*^2^ = 99.9.0%, *p* = 0.000), and the Egger test revealed no publication bias with a *p* value of 0.966 (Fig. 7).

### Sex of indexed child

**FIG. 8.**
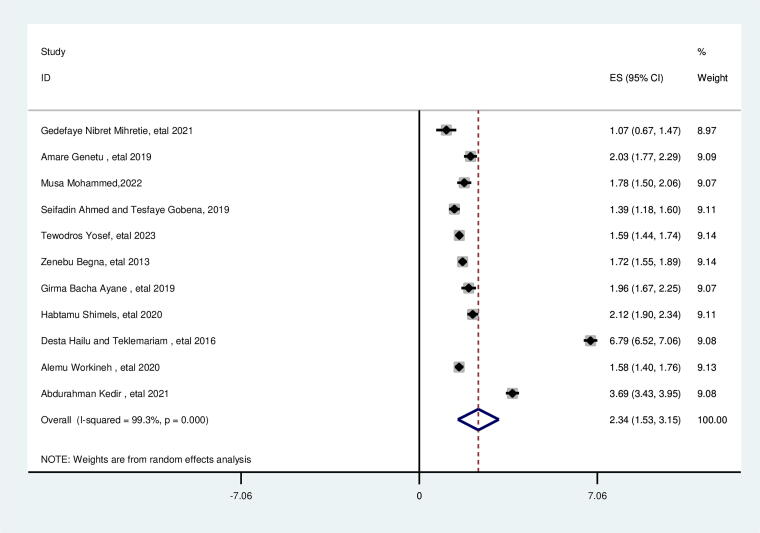
Pooled association between female sex of indexed child and suboptimal child spacing among reproductive age group women in Ethiopia.

In this review, the sex of the indexed child had a significant association with suboptimal birth spacing. The likelihood of suboptimal child spacing was 2.34 times higher for women whose index child were female than their counterparts (AOR = 2.34; 95% CI: 1.53, 3.15). This included the study’s heterogeneity (*I*^2^ = 99.3%, *p* = 0.000), and the Egger test identified no publication bias with a *p* value of 0.758 (Fig. 8).

### Survival status of indexed child

**FIG. 9.**
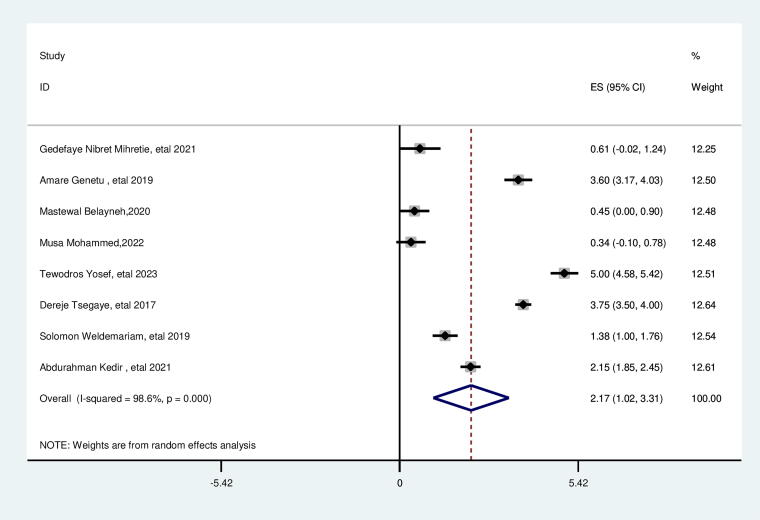
Pooled association between survival (death) of the index child and suboptimal child spacing among reproductive age group women in Ethiopia.

This review revealed a significant association between the survival status of indexed child and suboptimal birth spacing. The likelihood of suboptimal child spacing was nearly two times higher for women with nonsurvival index child than their counterparts (AOR = 2.17; 95% CI: 1.02, 3.31), with the heterogeneity of I2 = 98.6%, *p* = 0.000, and Egger test *p* value of 0.224, which showed no publication bias with non significant *P* value (Fig. 9).

### Publication bias

**FIG. 10.**
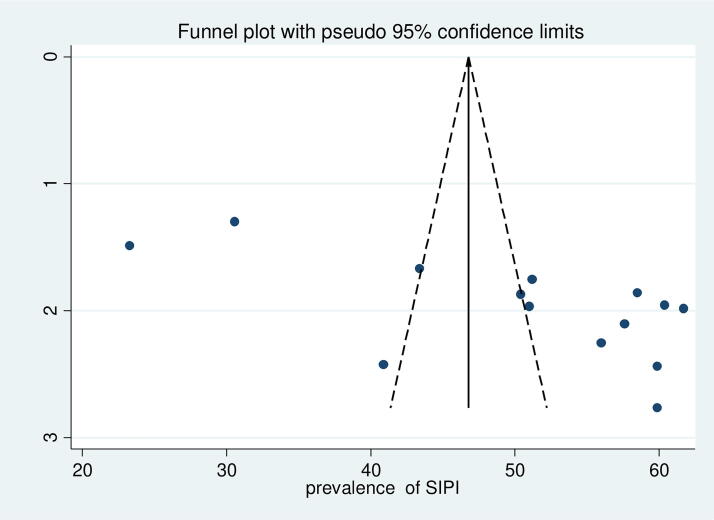
Funnel plot with 95% confidence limits of the pooled prevalence of suboptimal child spacing practice among reproductive age group women in Ethiopia.

The Egger test yielded a *p* value of 0.03 showing publication bias. Also, there is the asymmetrical distribution of included studies in the funnel plot, which suggests there is evidence of publication bias (Fig. 10). To adjust for the detected publication bias, a Trim and Fill analysis was conducted this helped to estimate the number of missing studies and adjust the overall pooled estimate. The analysis result showed no imputed or missing study, with no change in the result.

### Leave-one-out sensitivity analysis

**FIG. 11.**
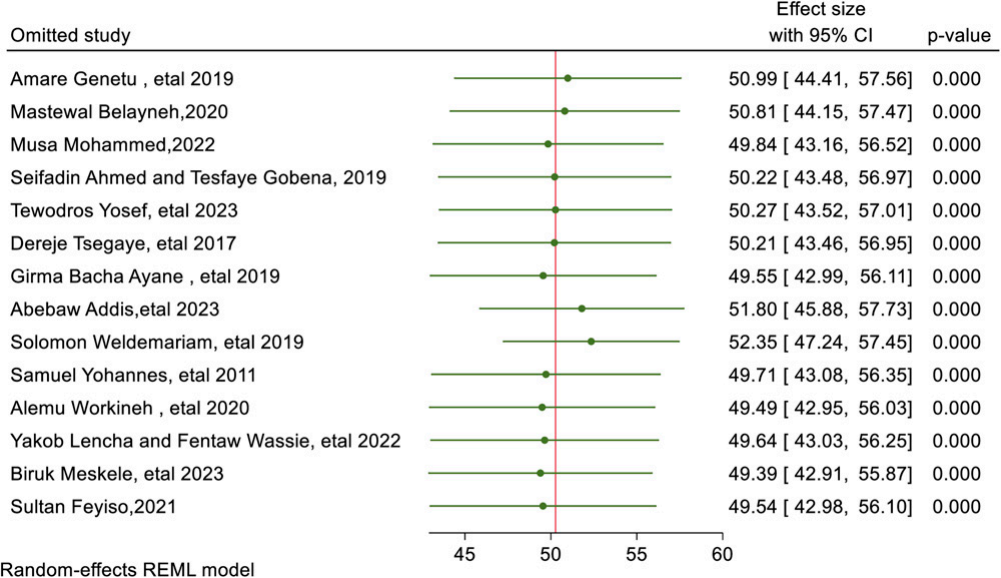
Sensitivity analysis of suboptimal birth spacing among reproductive age group in Ethiopia.

A leave-one-out sensitivity analysis was conducted to evaluate each study’s effect on the pooled prevalence of suboptimal birth spacing (SBI) by excluding each study step by step. The results showed that the excluded study did not significantly change the pooled prevalence (Fig. 11).

## Discussion

This systematic review and meta-analysis aimed to identify the pooled magnitude and factors associated with suboptimal birth spacing practice among reproductive age group women in Ethiopia. and revealed that the pooled prevalence of suboptimal birth spacing (SBI) among reproductive age group women was 50.29%, which is almost comparable with a study conducted in Ghana, Cameroon, and Uganda, which showed that the prevalence of SBIs, 46.9%,^55^ 49.7%,^25^ 46.10^4^, 52.4%^56^ respectively. However, this is higher than the study conducted at Kenya (20%),^23^ Tanzania (32.9%),^57^ rural and urban Nigeria (20.7% and 20.3%, respectively),^58^ Bangladesh (26.36%),^3^ Brazil (34.6%),^10^ Pakistan (22.9%),^2^ and Norway (10%).^59^ This might be explained by definition variation among different kinds of literature and lower than another study conducted in Ghana reported a SBI prevalence to be 80.0%,^60^ this might be explained by variation in fertility rates across country. The findings of this review were much higher when compared to those developed countries which showed 28.9% in the United States,^61^ European countries with the prevalence of SBIs ranging from 10%–15%,^62,63^ and in Australia and Canada with similarly low Prevalences of 10%–20%.^64,65^ This discrepancy may be attributed to factors such as limited access to modern contraceptive methods, inadequate family planning services, cultural norms that favor larger families, and lower levels of maternal education in developing countries. These challenges contribute to higher rates of SBIs, underscoring the need for comprehensive reproductive health interventions and increased educational efforts to improve maternal and child health outcomes in Ethiopia.

In this study place of residence, age of the mother at first marriage, and educational status of the woman were the sociodemographic factors that are associated with the practice of suboptimal birth spacing among reproductive-age women in Ethiopia. The odds of suboptimal birth spacing practice were 2.13 times higher for women living in rural areas than their counterparts. The factor of rural residency identified in our systematic review and meta-analysis is consistent with the findings of Antehunegn et al., who used data from the 2016 Ethiopian Demographic and Health Survey (EDHS). Although their study focused on a nationally representative sample, our analysis combines results from multiple studies, providing a broader understanding of how rural residency influences birth intervals. This alignment highlights the importance of rural residency as a key determinant of birth spacing practices across different data sources and study designs; this finding is also supported by a study done in Ghana.^25^ This might be explained by women living in rural have not much information on adverse outcomes of suboptimal birth spacing and may not have access to family planning services. Women who were married before the age of 18 had 1.94 times higher chance of suboptimal birth spacing than their counterparts and this finding is supported by studies done in Iraq and Pakistan,^2,66^ this might be explained by that women may have long fertile periods if they marry before the age of 18 than their counterparts since, the effective reproductive life span starts at the first menstruation or marriage, whichever is later. Another sociodemographic determinant of suboptimal birth spacing in this study was the educational status of the mother, which revealed that uneducated women had 3.39 times higher odds of suboptimal birth spacing practices than their counterparts. This is explained by the fact that women with no education may get married early, have more lives in rural areas, and may not know the adverse outcomes of SBIs.

This systematic review and meta-analysis identified that contraceptive usage is one of the determinants of suboptimal birth spacing practice among reproductive age group women, the odds of suboptimal birth spacing were 4.2 times higher among women who were nonusers of contraceptives than their counterparts. The factor of contraceptive usage identified in our systematic review and meta-analysis aligns with the findings of Antehunegn et al., who analyzed data from the 2016 EDHS. Although their study focused on a nationally representative dataset, our analysis synthesizes results from various studies, offering a more comprehensive understanding of how contraceptive use impacts birth intervals in Ethiopia. This consistency highlights the critical role of contraceptive usage as a determinant of birth spacing practices across different data sources and study methodologies. This is supported by studies conducted in Uganda,^67^ Cameroon,^4^ Pakistan,^2^ Iraq,^66^ and Malaysia.^1^ This might be explained by the fact that any method of family planning is designed to prevent pregnancy by different mechanisms and, on the contrary, if a reproductive-age woman is not using contraception, this might increase the prevalence of suboptimal birth spacing.

In this study, the odds of suboptimal birth spacing were 3.44 times higher for women who were breastfed their indexed child for less than 24 months than their counterparts and this finding is supported by studies done in Cameroon,^4^ Iraq,^66^ and Malaysia^1^ and might be explained by the fact that optimal breastfeeding, which is breastfeeding the child for at least 24 months, may prevent physiological preparation of the female reproductive tract for the next pregnancy, reduces fertility by prolonging postpartum amenorrhea, breastfeeding can reduce the probability that the next ovulation will result in conception and also in Africa, breastfeeding protects four births during a woman’s reproductive age, meaning she will have 1/3 fewer children than she might have otherwise.^68,69^

This systematic review and meta-analysis showed that the odds of suboptimal birth spacing were 2.34 times higher for women whose index child were female and 2.17 higher for women with nonsurvival index children than their counterparts this finding is supported by a study conducted in Uganda which stated A previous good fetal birth outcome and having a live child was negatively associated with SBIs,^67^ a study conducted in Iraq^66^ and Pakistan.^2^ This might be explained by shorter intervals being more likely to occur for women who have experienced child mortality because of the biological impact of subsequent infant death (the physiological alteration of pregnancy is short for women with death of neonate) or cultural and behavioral influences to replace the missing child.

## Conclusion

In this systematic review and meta-analysis, almost half of women give birth below the WHO recommendations of birth spacing which is 33 months and, in this review, rural residency, age of mother at first marriage, educational status of the woman, not using contraceptive, duration of breastfeeding, sex of indexed child and survival status of the indexed child were the main determinants of suboptimal birth spacing practice in Ethiopia. Increasing the utilization of contraceptives, avoiding early marriage, and practicing optimal breastfeeding for their baby at least for 24 months all may decrease the prevalence of suboptimal birth spacing practices in Ethiopia.

### Limitations and strengths of the study

As a strength, the included studies in this systematic review and meta-analysis were conducted both at health facilities and in the community as well as different designs (case-control, cross-sectional). As a limitation due to the use of a binary classification for birth intervals (below 33 months as “short” and above 33 months as “optimal”), we were unable to analyze birth intervals as a continuous variable or explore more detailed categories, which might have provided a more nuanced understanding of the data and also due to lack of studies in all regions of Ethiopia, this study may lack national representativeness.

## Data Availability

All related data and supporting information have been presented within the article.

## Abbreviations Used

AOR: adjusted odds ratio
CI: confidence interval
EDHS: Ethiopian Demographic and Health Survey
LMICs: low- and lower-middle incomes
SBI: short birth interval
