# Supplementary material for: The Magnitude and Determinants of Suboptimal Child Spacing Practices Among Women of Childbearing Age in Ethiopia: A Systematic Review and Meta-Analysis
Source: Womens Health Rep (New Rochelle). 2025 Mar 25;6(1):325–40. doi: 10.1089/whr.2024.0179 (PMC12040538; doi:10.1089/whr.2024.0179)
Supplement: Supplementary Data S2 [file whr.2024.0179_supplementary_data_s2.docx]

| Search engine | Search string | Total no of articles retrieved |
| --- | --- | --- |
| Google scholar | The Magnitude and Determinants of Suboptimal Child Spacing Practices Among Women of Childbearing Age in Ethiopia OR magnitude OR prevalence OR incidence AND determinants OR factors AND "Suboptimal Child Spacing" OR “short birth interval” OR “birth interval less than 33 months” AND practice OR Custom OR habit OR usage AND "Women of Childbearing Age "OR "fertile age" OR "reproductive age" OR "childbearing potential" OR "childbearing year" AND Ethiopia-meta-anlysis | 470 |
| PubMed | magnitude[All Fields] OR ("epidemiology"[Subheading] OR "epidemiology"[All Fields] OR "prevalence"[All Fields] OR "prevalence"[MeSH Terms]) OR ("epidemiology"[Subheading] OR "epidemiology"[All Fields] OR "incidence"[All Fields] OR "incidence"[MeSH Terms]) AND determinants[All Fields] OR factors[All Fields] AND "Suboptimal Child Spacing"[All Fields] OR "short birth interval"[All Fields] AND "practice"[All Fields] OR ("culture"[MeSH Terms] OR "culture"[All Fields] OR "custom"[All Fields]) OR ("habits"[MeSH Terms] OR "habits"[All Fields] OR "habit"[All Fields]) OR usage[All Fields] AND "Women of Childbearing Age "[All Fields] OR "fertile age"[All Fields] OR "reproductive age"[All Fields] OR "childbearing potential"[All Fields] OR "childbearing year"[All Fields] AND ("ethiopia"[MeSH Terms] OR "ethiopia"[All Fields]) | 1064 |
| Science Direct | Prevalence AND "Short birth interval" AND women AND Ethiopia | 44 |

S2 File: Search strategy and Output of search terms for the major databases.
