# Supplementary material for: The Magnitude and Determinants of Suboptimal Child Spacing Practices Among Women of Childbearing Age in Ethiopia: A Systematic Review and Meta-Analysis
Source: Womens Health Rep (New Rochelle). 2025 Mar 25;6(1):325–40. doi: 10.1089/whr.2024.0179 (PMC12040538; doi:10.1089/whr.2024.0179)
Supplement: Supplementary Data S3 [file whr.2024.0179_supplementary_data_s3.docx]

| Author | Selection Max 4 star | Comparability Max 2 star | Outcome Max 3 star | Score Max 9 star |
| --- | --- | --- | --- | --- |
| Gedefaye Nibret , etal 2021 | **** | * | *** | 8 |
| Amare Genetu ,etal 2019 | *** | ** | ** | 7 |
| Mastewal Belayneh,2020 | **** | ** | *** | 9 |
| Abebaw Addis,etal 2023 | **** | * | *** | 8 |
| Habtamu Shimels, etal 2020 | *** | * | *** | 7 |
| Musa Mohammed,2022 | *** | * | *** | 7 |
| Seifadin Ahmed and Tesfaye Gobena, 2019 | **** | * | *** | 8 |
| Tewodros Yosef, etal 2023 | *** | * | *** | 7 |
| Dereje Tsegaye, etal 2017 | *** | ** | ** | 7 |
| Zenebu Begna, etal 2013 | **** | * | *** | 8 |
| Girma Bacha Ayane , etal 2019 | **** | * | ** | 7 |
| Solomon Weldemariam, etal 2019 | *** | ** | ** | 7 |
| Samuel Yohannes, etal 2011 | **** | ** | *** | 9 |
| Desta Hailu and Teklemariam, 2016 | **** | ** | *** | 9 |
| Alemu Workineh,etal 2020 | **** | * | *** | 8 |
| Yakob Lencha and Fentaw Wassie, 2022 | *** | ** | *** | 8 |
| Biruk Meskele, etal 2023 | *** | ** | ** | 7 |
| Sultan Feyiso,2021 | *** | ** | *** | 8 |
| Abdurahman Kedir,etal 2021 | **** | * | *** | 8 |

S3 File: Newcastle-Ottawa Quality Assessment Scale of the included studies on the magnitude of sub-optimal child spacing practices and its associated factors among women of childbearing age in Ethiopia

^* Thresholds for converting the Newcastle-Ottawa scales to AHRQ standards (good, fair, and poor): Good quality: 3 or 4 stars in the selection domain AND1or2 stars in the comparability domain AND 2 or 3 stars in the outcome/exposure domain. Fair quality: 2 stars in the selection domain AND1or2stars in the comparability domain AND 2 or 3 stars in the outcome/exposure domain. Poor quality: 0 or 1 star in the selection domain or 0 stars in the comparability domain or 0 or 1 stars in the outcome/exposure domain.^
