# Supplementary material for: The Magnitude and Determinants of Suboptimal Child Spacing Practices Among Women of Childbearing Age in Ethiopia: A Systematic Review and Meta-Analysis
Source: Womens Health Rep (New Rochelle). 2025 Mar 25;6(1):325–40. doi: 10.1089/whr.2024.0179 (PMC12040538; doi:10.1089/whr.2024.0179)
Supplement: Supplementary Table S1 [file whr.2024.0179_supplementary_table_s1.docx]

| Factors | Amhara region (AOR) | Southern Region (AOR) |
| --- | --- | --- |
| Rural residency | 0.57 | 2.38 |
| Age at first marriage less than eighteen | 1.65 | 2.18 |
| Women with no formal educational status | --- | 3.55 |
| No contraceptive use | 7.76 | 3.73 |
| Duration of breastfeeding less than 24 months | 2.35 | 4.56 |
| The female sex of the index child | 1.76 | 4.18 |
| Survival (death) of the index child | 1.56 | --- |

Supporting Table 1: The Odds Ratios for Factors Associated with Short Birth Intervals in the Amhara and Southern Regions.
